# A Metabolomic Approach for the In Vivo Study of Gold Nanospheres and Nanostars after a Single-Dose Intravenous Administration to Wistar Rats

**DOI:** 10.3390/nano9111606

**Published:** 2019-11-12

**Authors:** Maria Enea, Ana Margarida Araújo, Miguel Peixoto de Almeida, Maria Elisa Soares, Salomé Gonçalves-Monteiro, Paula Guedes de Pinho, Eulália Pereira, Maria de Lourdes Bastos, Helena Carmo

**Affiliations:** 1 UCIBIO/REQUIMTE, Laboratory of Toxicology, Faculty of Pharmacy, University of Porto, 4050-313 Porto, Portugal; ana.margarida.c.araujo@gmail.com (A.M.A.); mematos25@gmail.com (M.E.S.); pguedes@ff.up.pt (P.G.d.P.); mlbastos@ff.up.pt (M.d.L.B.); 2 LAQV/REQUIMTE, Department of Chemistry and Biochemistry, Faculty of Sciences, University of Porto, 4169-007 Porto, Portugal; mpda@fc.up.pt; 3 LAQV/REQUIMTE, Department of Drug Sciences, Laboratory of Pharmacology, Faculty of Pharmacy, University of Porto, 4150-755 Porto, Portugal; salomegmonteiro@hotmail.com

**Keywords:** gold nanoparticles (AuNPs), shape, biodistribution, metabolomics, toxicity, in vivo

## Abstract

Gold nanoparticles (AuNPs) are promising nanoplatforms for drug therapy, diagnostic and imaging. However, biological comparison studies for different types of AuNPs fail in consistency due to the lack of sensitive methods to detect subtle differences in the expression of toxicity. Therefore, innovative and sensitive approaches such as metabolomics are much needed to discriminate toxicity, specially at low doses. The current work aims to compare the in vivo toxicological effects of gold nanospheres versus gold nanostars (of similar ~40 nm diameter and coated with 11-mercaptoundecanoic acid) 24 h after an intravenous administration of a single dose (1.33 × 10^11^ AuNPs/kg) to Wistar rats. The biodistribution of both types of AuNPs was determined by graphite furnace atomic absorption spectroscopy. The metabolic effects of the AuNPs on their main target organ, the liver, were analyzed using a GC-MS-based metabolomic approach. Conventional toxicological endpoints, including the levels of ATP and reduced and oxidized glutathione, were also investigated. The results show that AuNPs preferentially accumulate in the liver and, to a lesser extent, in the spleen and lungs. In other organs (kidney, heart, brain), Au content was below the limit of quantification. Reduced glutathione levels increased for both nanospheres and nanostars in the liver, but ATP levels were unaltered. Multivariate analysis showed a good discrimination between the two types of AuNPs (sphere- versus star-shaped nanoparticles) and compared to control group. The metabolic pathways involved in the discrimination were associated with the metabolism of fatty acids, pyrimidine and purine, arachidonic acid, biotin, glycine and synthesis of amino acids. In conclusion, the biodistribution, toxicological, and metabolic profiles of gold nanospheres and gold nanostars were described. Metabolomics proved to be a very useful tool for the comparative study of different types of AuNPs and raised awareness about the pathways associated to their distinct biological effects.

## 1. Introduction

Gold nanoparticles (AuNPs) are used for biomedical applications in diagnostic, drug therapy, and imaging [1,2,3,4]. Gold nanostars, containing multiple sharp tips, are particularly attractive in imaging and diagnostic, as compared with their spherical counter parts [5,6]. This anisotropic shape can lead to improved optical properties, but also to a different in vitro toxicological profile when compared with nanospheres [7,8]. Nevertheless, published studies are limited and fail to reach a robust and detailed description of shape-dependent biological effects of AuNPs [7,9]. Due to the low toxicity of AuNPs and to limitations of the experimental design (e.g., low tested concentrations, simultaneous variation of size, shape or coating, concentration expressed as gold mass versus number of nanoparticles) these studies highlight the lack of highly sensitive methods capable of detecting subtle differences to discriminate toxicity effects between different shapes. In a previous work of our group, with human cerebral microvascular endothelial cell line (hCMEC/D3), an in vitro model of the human blood-brain barrier (BBB), the use of molar concentration of AuNPs or Au mass concentration as a measure of dose for nanoparticles with different sizes yields different toxicological profiles [10]. The vast majority of studies of AuNPs effects in vitro and/or in vivo are analyzed using only Au mass concentration [8,10,11,12,13] overlooking the biological effects that depend on the amount of nanoparticles [9,14]. This emphasizes the need of new approaches that include analysis of exposure concentrations based on number of nanoparticles. 

Metabolomics has emerged as a useful tool for the toxicological study of drugs and can be applied for the biosafety evaluation of nanomaterials, providing a rigorous and comprehensive characterization of their toxicity [13,14,15]. Main advantages include the ability to identify and quantify a large number of metabolites in biological samples (fluids, cells, tissues) [16], providing detailed information about the metabolic pathways involved in the toxic response [15,17]. To our knowledge, the few studies of AuNPs effects on the metabolic profile use cell culture models (HepG2 and Caco-2 cell line) [13,14] or freshly isolated peripheral blood cells [18]. In the present work, AuNPs toxicity is evaluated by analysis of the metabolic profile of a target organ for nanoparticles toxicity in vivo. The liver is the main accumulation site of AuNPs after in vivo administration, as demonstrated by several studies [11,12,19] but the in vivo impact of the AuNPs on its metabolic pathways is still unknown. Therefore, to understand and discriminate the in vivo toxicity of two distinct types of AuNPs, our experimental design maintains all the relevant physical chemical properties (i.e., size and coating) constant except for shape, expressing the administered dose as number of gold nanoparticles per mL, to account for biological effects based on the amount of nanoparticles [10]. A gas chromatography-mass spectrometry (GC-MS)-based metabolomic study was performed to investigate the metabolic changes in the liver of the AuNPs-exposed animals, with comparative biodistribution and evaluation of conventional toxicological endpoints, like the levels of ATP and both reduced and oxidized glutathione.

## 2. Materials and Methods 

For a schematic representation of the experimental design, please refer to Figure S1 from the Supplementary Materials (Section 1. Experimental design).

### 2.1. Reagents and Materials

All reagents were of high purity or analytical grade. Some of the reagents (Nitric acid (HNO_3_), hydrogen peroxide (H_2_O_2_), palladium (II) nitrate [Pd(NO_3_)_2_], magnesium nitrate [Mg(NO_3_)_2_] and hydrochloric acid (HCl)) were purchased from Merck (Darmstadt, Germany) and all others from Sigma-Aldrich ((St. Louis, MO, USA). The AAnalyst 600 Atomic Absorption Spectrophotometer instrument and the gold pure calibration standard, used for Au quantification by GFAAS, were obtained from Perkin–Elmer (Shelton, CT, USA). An EVOQ 436 GC system (Bruker Daltonics, Fremont, CA, USA) was used for the chromatographic analysis.

### 2.2. Synthesis and Characterization of AuNPs 

The ~40 nm gold nanospheres were synthesized using a seed mediated growth method [20,21]. Briefly, in a first step, a seed suspension (~14 nm nanospheres) was obtained in water (150 mL total volume) by reduction of HAuCl_4_ (final concentration of 0.17 mM), at boiling temperature and under vigorous stirring, with 2.2 mM sodium citrate. Then the temperature was decreased to 90 °C, and the obtained seeds underwent successive growth steps by addition of 1 mL of 25 mM HAuCl_4_ until reaching the desired size (~40 nm). The suspension was left to cool. 11-Mercaptoundecanoic acid in ethanol (MUA, 10 mM) was added and the suspension was left overnight under vigorous stirring to exchange the coating from citrate to MUA. Then, the suspension was washed by three centrifugation steps [SIGMA 3-30K (Sigma, Osterode am Harz, Germany), 2000 g, 10 min, 25 °C] to eliminate excess of MUA, followed by resuspension of the pellet in phosphate buffer (10 mM, pH 7.4). 

Star-shaped gold nanoparticles were synthesized according to the method of Yuan et al. (2012) [22] by adding 450 µL of 50 mM HAuCl_4_, followed by simultaneously addition of 450 µL of 0.1 M ascorbic acid and 900 µL of 2 mM silver nitrate, under vigorous stirring (900 rpm), to 90 mL of aqueous seeds suspension (~14 nm nanospheres synthesized as previously described). The capping agent was exchanged by addition of 300 µL of 10 mM MUA in ethanol, followed by washing by centrifugation as described for spherical, and redispersion in phosphate buffer (10 mM, pH 7.4). 

Nanoparticle suspensions were sterilized by filtration using polyethersulfone Whatman membrane filters with 0.22 µm pore size (GE Healthcare Life Sciences, Chicago, IL, US). Stock solutions were kept at 4 °C, protected from light. No sign of aggregation was detected throughout the study. All experiments were performed using the same batches of gold nanostars and gold nanospheres. 

The synthesized AuNPs were characterized using a UV-Vis spectrophotometer Varian Cary 50 Bio (Agilent Technologies, Santa Clara, CA, USA), a Hitachi H-8100 transmission electron microscope (Hitachi, Tokyo, Japan), a NanoSight NS300 and a Zetasizer Nano ZS (Malvern Panalytical, Malvern, UK) for hydrodynamic size and zeta potential measurements. For detailed information regarding the methods used for AuNPs characterization, please refer to Supplementary Materials (Section 2. Characterization of AuNPs).

### 2.3. Animal and Experimental Design

All procedures were previously approved by the Ethics Committee of the Faculty of Pharmacy of the University of Porto. The experimental protocol used complies with the guidelines of the Committee on Care and Use of Laboratory Animal Resources (National Research Council, USA) and the European Communities Council Directive (86/609/EEC). 

Wistar rats (i3S, Porto, Portugal) with a body weight ranging from 200 to 250 g were randomly assigned to three groups (n = 5 each). Animals were anesthetized with ketamine:xylazine (40:5 mg/kg, i.p.) before slow injection of 2 mL/kg into the rat tail vein of either: (i) NaCl 0.9% (control), 1.33 × 10^11^ AuNPs/kg of (ii) Au nanospheres and (iii) Au nanostars. Gold nanoparticles were sonicated in an ultrasound bath (Bandelin Sonorex RK 100H; Berlin, Germany) for 10 min before administration, to obtain a uniform dispersion of the samples. The rats were maintained for 24 h in metabolic cages with a temperature and humidity-controlled environment, 12 h light/dark cycle, and food and water provided ad libitum. Over the 24 h exposure time, the animals were monitored for body weight, food and water intake, and urine and faeces were collected. Twenty-four hours after the single injection, the animals were sacrificed by exsanguination under anaesthesia (ketamine+xylazine 80:10 mg/kg, i.p.). Blood, faeces, urine, tissues, and organs including liver, spleen, lung, heart, kidney, brain, small intestine (duodenum), fat, skeletal muscle (quadriceps femuris) and tail were collected for gold determination by GFAAS. All organs were washed in 0.1 mM phosphate buffer, pH=7.4 and dried in blotting paper before further processing. The livers were partially used for further toxicological studies (ATP and glutathione levels) and for metabolomic investigation. The organ was weighted and divided into several parts after the washing procedure. The parts required for GC-MS metabolomics were immediately frozen in ice-cold isopropanol to stop the metabolism. The rest of the organ, necessary for GFAAS measurements and/or ATP and glutathione levels determination, were stored at −80 °C until analysis.

### 2.4. Determination of Gold in Biological Samples by Graphite Furnace Atomic Absorption Spectrometry (GFAAS)

The biodistribution profile of AuNPs in all the collected organs/tissues, whole blood, urine, and faeces was evaluated using a previously described GFAAS-based method [12]. (See Supplementary Materials for a detailed description). 

The results are presented as: (i) content of gold (Au ng/g organ) (ii) % of the injected dose, calculated from the GFAAS determination in organs/tissues versus injected dose; and (iii) estimated number of nanoparticles [AuNPs/g organ; as determined by combined Nanoparticle Tracking Analysis (NTA) and GFAAS techniques]. To estimate the results of the biodistribution study as the number of nanoparticles/g organ, the total content of gold was determined by GFAAS and the concentration in number of AuNPs/mL was determined by NTA for the stock suspensions [10]. Using the results from the two analysis, the average Au mass per nanoparticle was calculated. With these Au mass per nanoparticle values and the Au mass concentration/g organ, we were able to estimate the number of AuNPs/g organ. 

Normality of the data distribution was assessed by the Kolmogorov–Smirnov, D’Agostino & Pearson and Shapiro-Wilk normality tests. When data follow normal distribution, statistical comparisons between treatments were performed by the unpaired Student’s *t*-test. Otherwise, comparisons were made using the unpaired Mann-Whitney test. Significance was accepted at *p* values < 0.05. All statistical calculations were performed using GraphPad Prism software, version 6 (GraphPad Software, San Diego, CA, USA).

### 2.5. Intracellular Metabolome Analysis of the Liver

#### 2.5.1. Sample Collection and Preparation

Parts of the livers collected from the animals were rapidly frozen in an ice-cold isopropanol bath after previous washing (0.1 M phosphate buffer pH 7.4) and drying. All samples were kept at −80 °C until GC-MS analysis. The samples were thawed just before use. 

Distilled water was added to each sample (300 μL water per 20 mg organ weight) and further homogenized. Half of the same homogenized sample was used for the quality control samples (QCs) and the other half for GC-MS analysis. To all obtained aqueous suspensions, chloroform and methanol (1:1:3) were added and left for 30 min under moderate agitation. Afterwards, the samples were centrifuged for 10 min at 13,000× *g* at 4 °C and the supernatants collected into glass vials. Subsequently, 15 μL of internal standard (1mg/mL desmosterol) were added to each sample and the mixtures were vortexed for 1 min followed by evaporation to dryness at room temperature under a gentle stream of nitrogen. For the derivatization, the method of Chan et al was used [23]. Briefly, 50 μL of methoxyamine solution (15 mg/mL methoxyamine hydrochloride in pyridine) was added to the dried extract, incubated at 70 °C for 60 min followed by addition of 100 μL N,O-Bis(trimethylsilyl)trifluoroacetamide (BSTFA) with 1% Trimethylchlorosilane (TMCS) and incubated at room temperature for 60 min. The obtained derivatized residues were transferred to autosampler vials for GC-MS measurements.

In order to eliminate possible variations during sample collection and/or treatment, or resulting from the analytical procedure, QCs were used. They represent a mixture (at equal quantity) of all biological samples used in the study undergoing the same analytical processing as the samples.

The chromatographic and mass spectrometry settings used in this experiment were conducted according to a previously described method [17]. For the detailed information about the GC-MS settings, please refer to the Supplementary Materials (Section 4.1 Chromatographic and mass spectrometry settings).

#### 2.5.2. GC-MS Pre-Processing Data

The chromatograms obtained through GC-MS analysis were converted into an universal format (net.cdf file) using the MASSTransit version 3.0.1.16 (Palisade Corp, Newfield, NY) software. All data was pre-processed for baseline correction, peak detection, chromatogram deconvolution and alignment using the MZmine 2.21 software (parameters are displayed in Table S1 of the Supplementary Materials) and further normalized by total area. The data matrix was cleaned of artifact peaks such as GC contaminants (cyclosiloxanes, siloxanes, phthalates) by comparison with blanks, peaks with relative signal-to-noise ratio less than three, and of peaks with relative standard deviation (RSD) higher than 30% across all QCs.

#### 2.5.3. Multivariate Analysis

The data matrix was imported into the soft independent modelling of class analogy (SIMCA)-P 13.0.3 software (Umetrics, Umea, Sweden) to perform principal components analysis (PCA) and orthogonal partial least squares discriminant analysis (OPLS-DA) with the Pareto scaling method. The OPLS-DA loading S-plot allowed the identification of a number of variables with VIPs > 1 (variable importance to the projection), as being potentially responsible for the differences between groups (nanospheres versus control, nanostars versus control, nanospheres versus nanostars) [17,24]. Various parameters obtained by sevenfold cross-validation in the SIMCA-P 13.0.3 software were used to determine the quality of the models [24]. These parameters are the R^2^ (the fraction of the original data explained by the model), the Q^2^ (predictive capability of the model) and the CV-ANOVA p-value (analysis of variance testing of cross-validated predictive residuals) and values greater than or equal to 0.4 for the first two, and less or equal to 0.05 for CV-ANOVA were considered robust for discrimination purposes [25].

#### 2.5.4. Metabolites Identification Process 

The identification process of the metabolites was performed taking into consideration the retention time (RT), the retention index (RI), and the mass spectra from the obtained chromatograms. The RI was calculated using the RTs obtained from a solution of *n*-alkanes (C8-C40) for the same chromatographic column and temperature program. These parameters were compared with those from mass spectral libraries NIST14 (National Institute of Standards) and only matches equal or superior to 70% were considered. The discriminant metabolites were further analyzed using MetaboAnalyst 4.0 tool (GenomeCanada, Ottawa, Canada) to identify the corresponding biological pathways and to elucidate possible biological mechanisms [26].

#### 2.5.5. Univariate Analysis

For the univariate analysis of a specific discriminant metabolite among groups, the corresponding variables with VIPs > 1 as shown by the multivariate analysis were taken into consideration [17]. The metabolites were statistically analyzed between different groups (nanospheres, control, nanostars) using GraphPad Prism version 6 (GraphPad software, San Diego, CA, USA) by the Shapiro-Wilk normality test, the unpaired Student´s t test (data normally distributed) and the unpaired Mann-Whitney test (data non-normally distributed). Significance was considered at *p* value < 0.05.

### 2.6. In Vivo Toxicological Evaluation of AuNPs

The parts of liver for the toxicological evaluation were firstly dispersed in 0.1 M phosphate buffer pH 7.4 (1:4 m/v) and homogenized using an Ultra-Turrax® homogenizer (Ystral, Ballrechten-Dottingen, Germany). The resulting suspension was diluted with 5% HClO_4_ and further centrifugated at 13,000× *g*, at 4 °C, for 10 min. The obtained supernatants were stored at −80 °C for the quantification of ATP and GSH, while the pellets were dispersed in 0.3 M NaOH and used for protein quantification using the Lowry’s method [27]. Briefly, to 50 μL of neutralized pellet (sample), standard, or blank (just buffer without sample), 100 μL of reagent A (14.7 mL of 2% Na_2_CO_3_, 0.15 mL of 2% KNaC_4_H_4_O_6_·4H_2_O, and 0.15 mL of 1% CuSO_4_·5H_2_O) was added. After 10 min incubation in the dark, at room temperature, 100 μL of reagent B (Folin and Ciocalteu’s phenol reagent, diluted 1:15 with distilled water) was added. After incubation for 20 min, under the same conditions, the absorbance was measured at 750 nm in a 96-well microplate reader (PowerWaveX; Bio-Tek Instruments, Winooski, VT, USA). All samples were tested in triplicate. The blank and the protein standards (with bovine serum albumin BSA) were prepared in 0.3 M NaOH. 

For ATP determination, 150 µL of the homogenized supernatant was neutralized with the same volume of 0.76 mM KHCO_3_ and centrifuged (13,000 rpm, 10 min, 4 °C). The ATP intracellular level was quantified in the neutralized supernatant using a luciferin/luciferase bioluminescent method as previously described [28]. Briefly, 75 µL of luciferin–luciferase reagent (0.15 mM luciferin; 300,000 light units of luciferase from Photinus pyralis (American firefly); 50 mM glycine; 10 mM MgSO_4_; 1 mM Tris; 0.55 mM EDTA; 1% BSA; pH 7.6; 4 °C; protected from light) was added to 75 µL of neutralized supernatant just before luminescence readings. Luminescence was measured in a multi-well plate reader (BioTek Synergy^TM^ HT, Bio-Tek Instruments, Winooski, VT, USA) set to 560 nm emission. 

Both total glutathione (GSHt) and oxidized glutathione (GSSG) were quantified using a previously described method [28] of the DTNB-GSSG reductase recycling assay. For the GSSG determination, 10 μL of vinylpyridine was added to 200 µL of all samples, standards and blank, and further shaken during 1 h on ice. Then, 2-vinylpyridine-treated samples (GSSG) and homogenized supernatants (GSHt) were neutralized with 0.76 mM KHCO_3_ and further centrifuged as described for the ATP assay. Then, 65 μL of fresh reagent solution containing 1.58 mg/mL DTNB and 0.57 mg/mL β-NADPH were added to 100 µL of the supernatant, left for 15 min incubation at 30 °C in the dark, and followed by the addition of 40 μL of glutathione reductase solution (10 U/mL). The reaction was monitored for 3 min, at 415 nm, in a multi-well plate reader and compared with a standard curve. The amount of GSH was calculated by subtracting GSSG from the GSHt: GSH = GSHt − (2 × GSSG). For ATP, GSHt, and GSSG determinations, blank and standards were prepared in 5% HClO_4_ and the results were normalized to total protein content (nmol/mg protein).

### 2.7. Statistical Analysis of the Biochemical Parameters

Results of GSH/GSSG and ATP assays are presented as mean ± standard error of the mean (SEM). Normality of the data distribution was assessed by the Kolmogorov–Smirnov, D’Agostino & Pearson and Shapiro-Wilk normality tests. When data followed normal distribution, statistical comparisons among groups were done using one-way analysis of variance (ANOVA) followed by Tukey’s multiple comparison test. Otherwise, comparisons were made using the Kruskal–Wallis test followed by the Dunn’s post hoc test. Nanospheres versus nanostars values were compared by the unpaired Student’s *t*-test. Overall, significance was accepted at *p* values < 0.05. All statistical calculations were performed using GraphPad Prism software, version 6 (GraphPad Software, San Diego, CA, USA).

## 3. Results

### 3.1. Synthesis and Characterization of AuNPs

**Figure 1:**
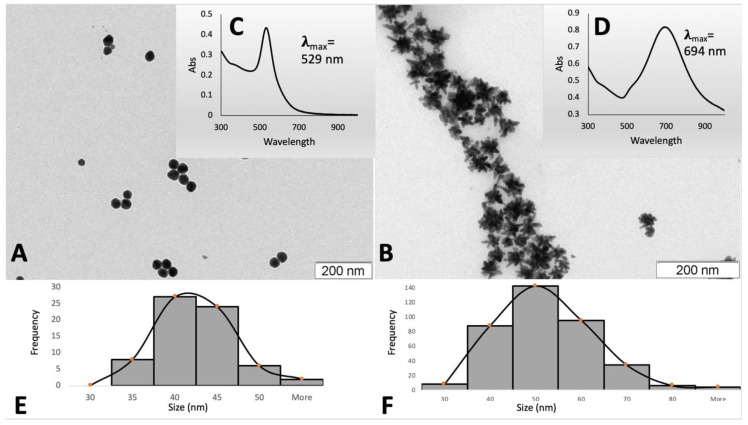
Representative TEM images of the synthetized gold nanospheres (**A**) and gold nanostars (**B**). The insets show UV-Vis spectra (**C**,**D**) and size distribution by frequency resulting from TEM analysis (**E**,**F**).

MUA-coated gold nanospheres (40 nm) and gold nanostars (approx. 47 nm) were synthesized and characterized using UV-Vis spectrometry and transmission electron microscopy, TEM (Figure 1). 

Table 1 summarized the main physicochemical properties of the synthesized AuNPs. The average size of the samples as measured by TEM was 40.0 ± 4.6 nm for nanospheres and 47.4 ± 10.7 nm for nanostars. Hydrodynamic diameters determined by dynamic light scattering, DLS, (52.6 ± 0.1 nm for nanospheres and 64.4 ± 0.3 nm for nanostars) and nanoparticle tracking analysis, NTA, (59.1 ± 2.5 nm for nanospheres and 60.0 ± 1.5 nm for nanostars) were slightly higher. Gold concentrations of the stock suspensions of gold nanoparticles were determined by GFAAS (72.78 ± 4.17 µg/mL for nanospheres and 180.33 ± 13.76 µg/mL for nanostars), while AuNPs concentration was determined by NTA [(6.68 ± 0.08) × 10^10^ particles/mL and (18.2 ± 0.8) × 10^10^ particles/mL respectively]. Both nanospheres and nanostars are negatively charged due to the presence of MUA on the surface of AuNPs, as shown by the zeta potential from DLS measurements (Table 1).

### 3.2. Influence of Shape in the Biodistribution Profile of AuNPs

The biodistribution patterns for gold nanospheres and nanostars, after 24 h of the single-dose administration to rats, are presented in Table 2.

Au nanospheres were distributed mainly in the liver (3648 ± 329 ng/g), followed by the spleen (2624 ± 329 ng/g) and the lung (31.7 ± 7.0 ng/g) (*p* < 0.05 liver versus spleen, spleen versus lung, liver versus lung). For Au nanostars, no statistical differences were found between liver (2697 ± 205 ng/g) and spleen (2271 ± 202 ng/g) accumulation, but a significantly lower content was found in the lung (44.0 ± 6.7 ng/g) as compared to liver and spleen (*p* < 0.001). For both Au nanospheres and nanostars, Au content in the heart, kidney, urine and blood was always lower than the limit of quantification (LOQ = 4.12 ng/g).

Regarding the shape-dependent biodistribution profile, differences between the two types of AuNPs were noted. Au content in the liver of animals treated with gold nanospheres was significantly higher in comparison to those treated with gold nanostars (*p* < 0.05). However, this significance was lost when the data are presented as % of the injected dose or expressed in number of AuNPs/g. In the spleen, no significant difference was found between the two morphologies. The biodistribution in the lung also seems to be shape-independent both regarding Au content and % of the injected dose, as no differences were found between the two morphologies. Nevertheless, different content in AuNPs with a slightly higher content in AuNPs/g organ for gold nanostars as compared with nanospheres (*p* = 0.0532) was found. Therefore, biodistribution data must be interpreted with caution since the results depend on how the concentration is expressed.

### 3.3. Quality Evaluation of the Metabolomic Study

**Figure 2:**
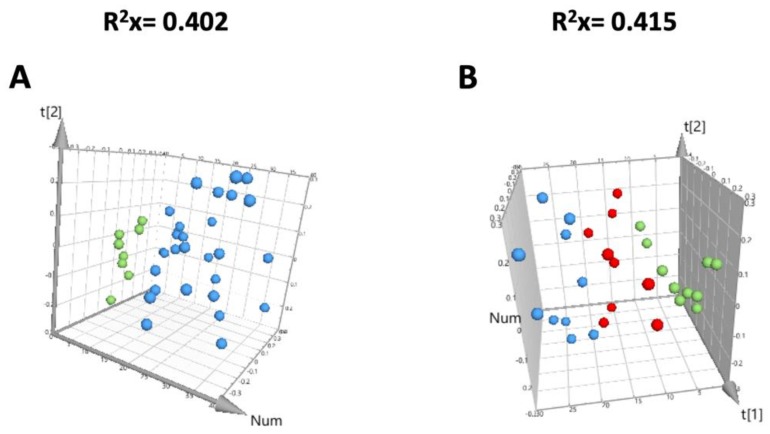
3D principal components analysis (PCA) score scatter plot obtained for the liver chromatograms corresponding to QCs group (green dots) versus treated group represented in blue dots (with 0.9% NaCl, with nanospheres or nanostars) (**A**) and three group analysis corresponding to the nanostars-treated group (blue dots) versus nanospheres-treated group (red dots) versus the control group (green dots) after analysis of the metabolome (**B**).

**Figure 3:**
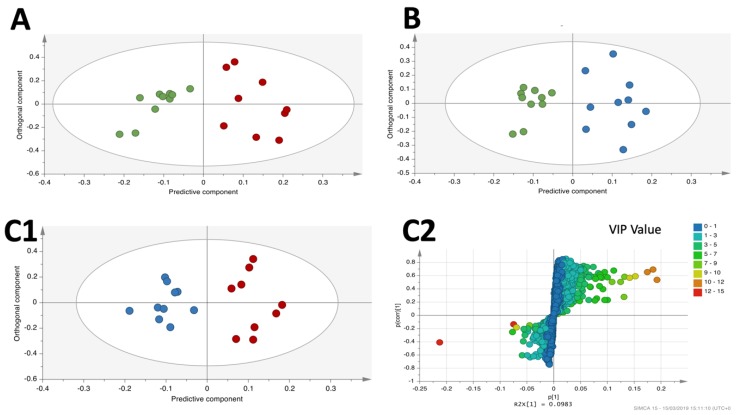
OPLS-DA score scatter plot obtained for the liver chromatograms corresponding to (**A**) nanospheres-treated group (red) versus control group (green); (**B**) nanostars-treated group (blue) versus control group (green); (**C1**) nanospheres-treated (red) versus nanostars-treated group (dots); and (**C2**) the corresponding OPLS-DA S-plots obtained for the respective pairwise comparison with the variables colored according to their VIP value.

Quality assessment of data obtained by GC-MS was the first step done in the metabolomic analysis. In order to check for reliability and high-quality, we used several quality controls (QCs) that were clustered in the 3D PCA scores plot, as seen in Figure 2A. An outlier was found in the liver data (nanospheres group) since one sample was outside the Hotelling´s T2 region in the PCA score plot. This outlier was attributed to technical chromatographic issues. As depicted in the PCA score plot for liver (Figure 2B), samples show a tendency to cluster into three groups from the metabolomic analysis (negative control, nanosphere-treated and nanostar-treated animals). In order to maximize the separation between groups and to find the significant discriminant metabolites, in addition to unsupervised analysis (PCA), we performed an OPLS-DA analysis as detailed below (Figure 3: A, B and C1). Using this supervised pairwise analysis, from all GC-MS detected metabolites in the liver, the following potentially discriminants (VIP > 1) were identified: (i) nanospheres versus control treated animals—57 metabolites; (ii) nanostars versus control treated animals—66 metabolites and (ii) nanospheres versus nanostars treated animals—57 metabolites. A list of these potential metabolites with their general characteristics, such as retention time (RT), characteristic ions (m/z), calculated (RIcalc) and theoretical (RIlit) retention indexes, reverse match factor, and identification method is presented in Table S2 in Supplementary Materials. The identification of the hepatic metabolites was done using analytical standards (for 22 metabolites) and by spectra similarity with commercially available spectral libraries (40 metabolites), while 5 of the metabolites were not yet identified (Table S2 of the Supplementary Materials).

### 3.4. OPLS-DA Liver Analysis Clearly Discriminated the Metabolic Profile of Nanospheres and Nanostars Compared to Control

OPLS-DA analysis proved that there is a clear distinction between AuNPs treatment (nanospheres and/or nanostars) versus control (Figure 3A and Figure 2B). The corresponding OPLS-DA parameters (Q^2^ and *p* values) for each of these comparisons have good values, as depicted in Table 3. To validate the OPLS-DA models, corresponding permutation tests for each OPLS-DA model were performed and, as expected, their R^2^ and Q^2^ values (Table 3) were lower than for the original classes.

Special attention should be given to Q^2^ higher than 0.4 for all comparisons (nanospheres versus control, nanostars versus control) and *p* values lower than 0.05, indicative of a good discrimination between classes. Taking into consideration the OPLS-DA loading S-plot for the liver, the potential discriminatory liver metabolites for all three comparisons with the VIPs > 1 were integrated. 

For the comparison of nanospheres versus control, among 57 liver metabolites considered important for discrimination (VIPs > 1), 10 were considered statistically significant (*p* < 0.05), 3 presented *p* values between 0.05 and 0.11 (Table 4) and the remaining (44 metabolites) have no statistical significance (*p* > 0.11). For the nanostars versus control comparison, among the 66 metabolites considered important, 12 were statistically significant (*p* < 0.05); and 8 presented values between 0.05 and 0.11. The remaining metabolites contributed to the discrimination process but without significant *p* values (the complete list of all metabolites can be found on Table S2 of the Supplementary Materials).

Most of the metabolites that were considered important (*p* values < 0.11) in the discrimination process belong to the class of fatty acids. Metabolites such as oleic, palmitic and linoleic acids were found increased in both nanosphere- and nanostar-treated animals (versus control) while others, such as palmitoleic, docosahexaenoic, myristic, arachidonic, and 5,8,11-eicosatrienoic acids, were increased only in the nanosphere-treated animals. Other metabolites found altered in both AuNPs treatments belong to the class of the nucleosides/nucleotides: inosine and uridine decreased, while uracil increased in treated samples. In both nanosphere and nanostar-treated animals glyceric acid was found to increase. 

Other metabolites belonging to the class of organic acids and derivatives were altered only in the nanostars-treated animals. Glycerol, propanoic acid and urea were found increased while squalene and pyrogallol decreased. Metabolites from the class of aminoacids, such as L-lysine, dimethylglycine, L-isoleucine, L-proline, L-serine, L-threonine, were found increased also only in the nanostars-treated animals (versus control). A decrease in myoinositol and an increase in D-Psicofuranose, a metabolite from the group of sugars, was noticed in the nanostars-treated animals. Metabolites belonging to the class of inorganic acids were also found altered, with a decrease in phosphoric acid and an increase in boric acid.

### 3.5. OPLS-DA Liver Analysis Clearly Discriminated the Metabolic Profile of Nanospheres versus Nanostars

OPLS-DA analysis of nanospheres versus nanostars (Figure 3C1) and the corresponding Q^2^ (0.568) and *p* value (0.0139) (Table 3) shows a good separation between the two classes reinforced by the lower R^2^ and Q^2^ values of the corresponding permutation tests.

Taking into consideration the S-plot for the nanospheres versus nanostars comparison (Figure 3C2), 57 distinct metabolites were considered important for discrimination (VIPs > 1). Among them, 14 were statistically significant (*p* < 0.05) and 7 had *p* values between 0.05 and 0.11 (Table 4), whilethe remaining were non-significant (*p* values > 0.11).

Many of the previously described metabolites proved to be statistically significant and discriminated between nanospheres and nanostars. All of the previously mentioned fatty acids (Table 4), with the exception of myristic acid, demonstrated to be important in the distinction of nanosphere- versus nanostar- treated animals. Other metabolites considered important belong to various chemical groups such as: organic acids and derivatives (maleic acid, pyrogallol), inorganic acids (phosphoric and boric acid), benzene and substituted derivatives (o-ethyltoluene), amino-acids and derivatives (L-lysine, dimethylglicine) or nucleoside/nucleotide (inosine, uridine, uracil). Only 3 metabolites remained unknown (Unknown 1, Unknown 2, Unknown 3) (Table 4).

### 3.6. In Vivo Toxicological Evaluation of AuNPs

**Figure 4:**
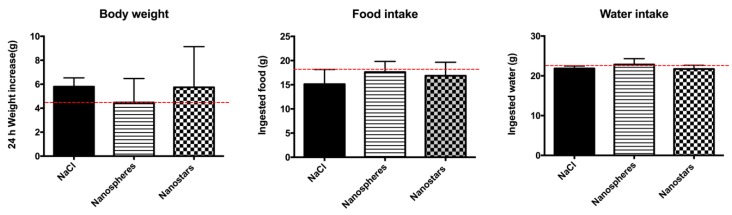
Graphical representation of body weight variation (**A**), food (**B**) and water intake (**C**) of the Wistar rats after 24 h i.v. treatment with 2 mL/kg of 0.9% NaCl solution (control), 10 mM phosphate buffer pH 7.4 (solvent) or Au nanospheres (spheres) or Au nanostars (stars) in concentration of 1.33 × 10^11^ AuNPs/kg animal. Results are expressed as mean ± SEM (n = 5. The data was analyzed with Kolmogorov–Smirnov, D’Agostino & Pearson and Shapiro-Wilk tests in order to check for normal distribution. For statistical comparisons between groups, one-way analysis of variance (ANOVA) followed by Tukey’s multiple comparison test were performed. Solvent versus negative control and spheres versus stars values were compared by the unpaired Student’s *t*-test. Significance was accepted at *p* values < 0.05.

The rats tolerated well the i.v. injection of AuNPs and did not exhibit any behavioral changes during the study. The macroscopic analysis did not reveal any signs of inflammation at necropsy for all four groups. No differences were found in body weight variation, food and water intake between AuNPs-treated animals and the control (0.9% NaCl) (Figure 4). 

**Figure 5:**
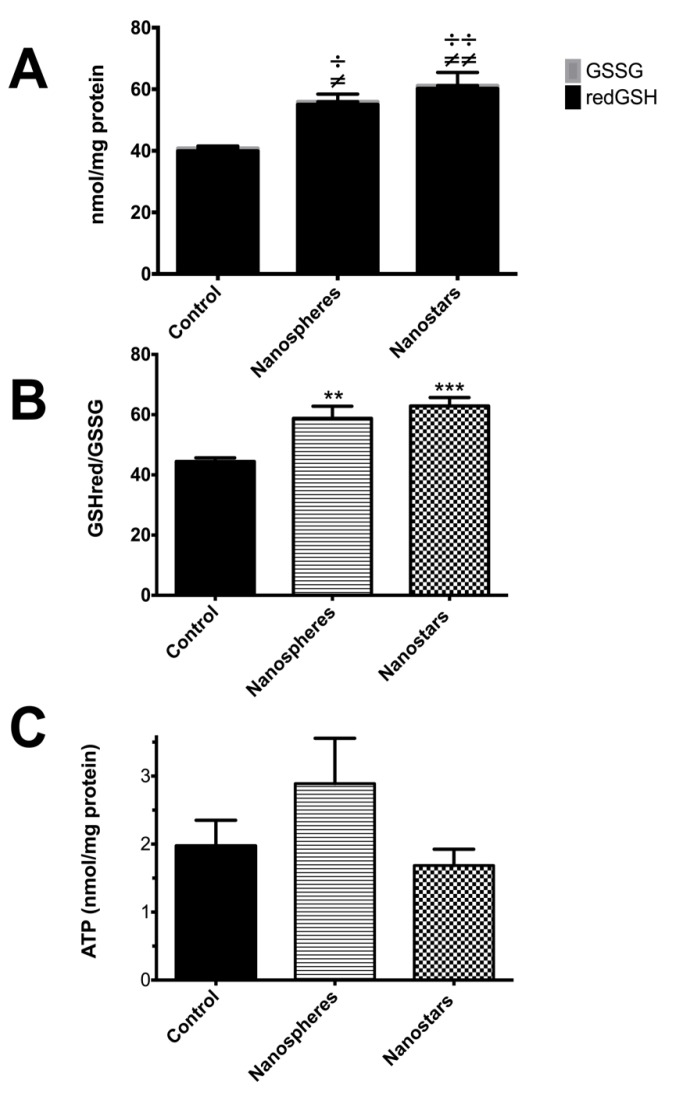
Hepatic GSSG, GSH and GSHt (**A**), GSH/GSSG ratio (**B**) and ATP (**C**) levels of animals treated with nanospheres, nanostars, solvent, and control. Results are expressed as mean ± SEM (n = 5). Data was assessed by the Kolmogorov–Smirnov, D’Agostino & Pearson and Shapiro-Wilk tests for normal distribution. For the statistical comparisons between groups, one-way analysis of variance (ANOVA) followed by the Dunn’s post hoc test was performed, except when data did not follow normal distribution. In this case, the Kruskal–Wallis test followed by the Dunn’s post hoc test was used. Solvent versus negative control and nanospheres versus nanostars values were compared by the unpaired Student’s *t*-test. (**A**) ≠ *p* < 0.05, ≠≠ *p* < 0.01 versus negative control for GS; ÷ *p* < 0.05, ÷÷ *p* < 0.01 versus negative control in GSHt determination; (**B**) ** *p* < 0.01 versus control group for GSH/GSSG ratio).

There was an increase in the hepatic GSH levels of the AuNPs-treated animals versus controls, for both spherical (*p* < 0.05) and star-shaped nanoparticles (*p* < 0.01), as well as for the GSH/GSSG ratios, resulting in an increase in the total glutathione levels (*p* < 0.05) but without influencing the oxidized glutathione. ATP levels remained unchanged (Figure 5).

## 4. Discussion

In the present study, gold nanospheres and nanostars that have shown to be promising for biomedical applications [1,5] were synthetized and characterized. The characterization data agreed well with the literature [20,22,29]. Star-shaped AuNPs show better optical properties than nanospheres of similar diameters [1,5], but the question remains whether these improved optical properties also implicate significant differences in the way star-shaped nanoparticles interact with biological systems. Even if AuNPs of approximately 50 nm diameter were described as noncytotoxic in phagocytic and nonphagocytic hepatic cells, a shape-dependent toxic effect was described in other cells [10,30]. In fact, several in vitro studies have shown differences in toxicity and uptake, but the conclusions were contradictory [7,9]. For example, Favi et al. reports that 61.46 nm gold nanospheres are more toxic than 33.69 nm gold nanostars on rat fat endothelial cells, while in Sultana et al. study, 50 nm gold nanostars are more cytotoxic than 50 nm gold nanospheres to human endothelial cells [7,9]. An additional concern with these in vitro studies is the lack of in vivo complexity and specially the relevance of the low but biologically relevant in vivo doses [8,31,32]. In addition, the use of gold nanoparticles with similar physicochemical properties is very important in comparison studies, as the simultaneous variation of different properties (e.g., size, shape, or coating) can easily confound data interpretation [7,8,31]. In the present study, in order to facilitate interpretation, we used appropriate synthesis methods [20,22] to guarantee a similar diameter, and used the same coating agent. 

A likely confounding factor that explains the contradictory results often found in comparative studies is the way the dose is calculated and expressed in the experimental study design. The vast majority of studies use Au mass per animal weight as dose, which represents the gold mass concentration as determined by GFAAS or inductively coupled plasma mass spectrometry (ICP-MS) [11,33,34]. But, as previously demonstrated, the same Au mass concentration does not necessarily translate into the same concentration of gold nanoparticles, leading to inaccurate comparative studies [10,35,36]. This is especially relevant when comparing nanoparticles of different sizes, for which Au mass concentration versus AuNPs concentration varies the most [10,36]. In the present study, we were able to synthesize nanospheres and nanostars of very similar sizes (~40 nm versus ~47 nm, respectively), but still decided to calculate the administrated dose in nanoparticle concentration to avoid misleading results. As expected, the same concentration administrated in AuNPs (1.33 × 10^11^ AuNPs/kg) did not correspond to the same concentration in Au mass (146 µg/kg for nanospheres and 120 µg/kg for nanostars). We therefore believe that, with this study design, we can accurately compare two types of nanoparticles, at the same tested dose, and with all relevant physical chemical properties kept constant except for the shape. 

The biodistribution of the tested nanoparticles was shape-dependent, and the liver and the spleen were, as expected, the main site for accumulation for both types of AuNPs [12,34]. A higher gold content was found in HepG2 cells exposed to nanospheres as compared to nanostars [37], which agrees well with our in vivo data. The uptake of AuNPs seems to be, not only shape-dependent, but also dependent on the cell/organ type, as distinct biodistribution profiles were determined for the liver, spleen, and lung. In vitro studies have previously shown that, at same tested Au concentration, gold nanospheres are internalized to a higher extent than nanostars in particular cell lines [32,37], while for other cell types the opposite occurs [9,31]. A content in Au lower than the LOQ found in the other organs, tissues and fluids was probably due to the lower dose of injected AuNPs compared with other in vivo studies [11,12]. Nevertheless, a lower content in Au for these organs, tissues and fluids compared with liver, spleen or lung was previously reported for similar experimental conditions [11,12]. The lack of Au content in brain was expected since in a previously published paper of our group, ~50 nm MUA-coated nanospheres and nanostars were unable to cross the in vitro hCMEC/D3 human blood brain barrier model [10].

At the tested Au concentration, the animals did not show any behavioral change upon exposure to various treatment groups versus control. Nevertheless, severe changes in behavior, hypopnea, tremor and arching of the back and even death were already reported in animals treated with other types of AuNPs, such as rods and nanocages at high doses, and with 40 nm gold nanospheres. For the 40 nm gold nanospheres, the effects were scarcer than for gold nanorods, suggesting a shape-dependent effect, and it was only observed at the highest dose of ~10^12^ AuNPs/g animal [38]. So, the effect was also concentration-dependent and the lack of any behavioral change from our study can be easily explained by the lower concentration in AuNPs administrated to the rats. In fact, the concentration of AuNPs used in our study was lower than in many vivo studies [11,12,38], so no significant differences are expected in the conventional toxicological assays. As metabolomics showed the ability to detect modifications in various biological pathways even at low subtoxic concentration of AuNPs [39], further discussion will mainly focus on this approach. 

Variables associated to metabolic data due to collection, storage and preparation of the samples either by incorrect data processing or equipment operation can affect the quality of the analysis [40]. In order to eliminate this variability, quality control samples (QCs) corresponding to a pool of all samples included in the study were used [40]. The relative standard deviation (RSD) of each variable across the QCs was used as an indicator of the reproducibility and repeatability of the analysis [40]. The unsupervised multivariate analysis method, represented by a principal component analysis (Figure 2) showed that QCs cluster in the score plot separates from the treated group, suggesting a high data quality. 

For the unsupervised multivariate analysis of the metabolomic fingerprinting, PCA is the most widely used method [25,41]. Normally, is the first line analysis to formulate initial biological hypothesis that are further confirmed using a supervised method, such as orthogonal partial least squares discriminant analysis (OPLS-DA) [25,42]. An OPLS-DA classification guided by well-separated PCA scores has a greater likelihood of producing biologically relevant results. In the current study, PCA showed a tendency to separate among all groups. OPLS-DA analysis, recommended for two class separation, was further applied to maximize this separation tendency and to obtain a clearer and straightforward interpretation [42]. By using OPLS-DA analysis, the variation within group (for example animal variation) is usually eliminated, resulting in much better scores in which classes are separated. One of the challenges of using OPLS-DA analysis is the possibility of presenting class separation even for a completely random variable [25], so further validation is necessary. The current study obtained good class separation for the liver analysis (Figure 3) of Au nanospheres versus Au nanostars. In order to validate the results obtained by OPLS-DA in the liver, among many cross-validation parameters (Table 3), the quality assessment statistic (Q^2^) that indicates how well the model predicts new data is often recommended [40]. Values higher than 0.4 obtained for the liver samples are considered acceptable [25]. Another validation was made using CV-ANOVA analysis, and the significant results for the liver with *p* < 0.05 reinforce the quality of the model [41] (Table 3). The sum of squares of uncorrelated (R^2^X) and predictive (R^2^Y) components representing the variation explained by the model are also used for validation [25,40]. High values of R^2^ and Q^2^ were described as good indicators for the power of models in selecting significant data [25,40]. As suggested, OPLS-DA can lead to a good separation even when data are randomly distributed, as the permutation tests were used to randomly assign class labels to different individuals [43]. In order to evaluate whether the specific classification of the individuals in the two designed groups (OPLS-DA) is significantly better than any other random classification in two arbitrary groups, the classification models of the permutation test are further calculated and the obtained values (R^2^ and Q^2^) should be lower than the initial OPLS-DA analysis [40,43]. The performed permutation tests for the liver show lower values than in the original OPLS-DA analysis, as required for validation (Table 3).

**Figure 6:**
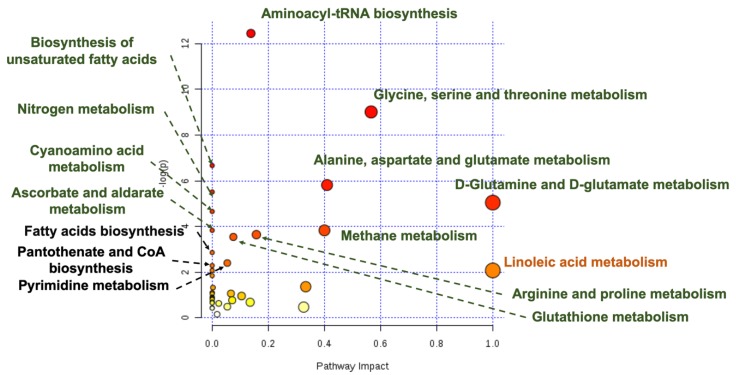
Graphical representation of liver altered metabolic pathways as obtained from MetaboAnalyst 4.0 tool [26]/taking into consideration the discriminant metabolites between nanospheres and nanostars with VIPs > 1; pathways with green letters have *p* value < 0.05, black: *p* value < 0.1 and yellow/orange *p* > 0.1.

Finally, discriminant metabolites obtained from the GC-MS analysis should be associated to biochemical processes to get the big picture of all metabolic changes that characterize a specific treatment [40]. Many metabolites can be common to specific biological pathways and software such as the MetaboAnalyst can be used to integrate all metabolites in specific biochemical pathways [17]. In fact, biological pathways as the metabolism of glutathione, ascorbate, fatty acids, aminoacids, and purine and pyrimidine, were found altered in both nanospheres- and nanostars-treated animals compared to control and were considered also important in the comparison between gold nanostars and nanospheres (Figure 6). These metabolic pathways were previously related to the toxicological effect of AuNPs [44].

Some of the discriminant metabolites, such as pyrogallol and dimethylglycine, were associated to the production of reactive oxygen species (ROS). Pyrogallol can produce oxidative damage and subsequently mutagenesis, carcinogenesis, and hepatotoxicity [45] while dimethylglycine, an intermediate of choline oxidation, is considered an effective ROS generator (of O_2_^•−^ and hydrogen peroxide in liver mitochondria, via electron transfer to Complex 1), which can further lead to lipid peroxidation and cell injury [46]. The involvement of AuNPs in ROS production and subsequent oxidative stress has also been previously reported [44,47]. The cellular responses to ROS attack can manifest through (i) increasing de novo synthesis of GSH and/or (ii) GSH oxidation. In this study, no significant increase was noticed for glutamine, glycine and cysteine, precursors of GSH, for none of the tested conditions. In accordance, ATP levels were not altered by the biochemical assays, contrary to what would be expected in de novo synthesis of GSH, that requires high energy levels [48,49]. On the other hand, GSH oxidation normally occurs in the presence of selenium-dependent GSH peroxidase, with the production of GSSG which is further reduced back to GSH by GSSG reductase in the presence of NADPH, together with enzymes as glutathione reductase and glutathione peroxidase [50]. Therefore, the results suggest that the responsible for the increase of GSH, as in the current study, is not de novo synthesis of GSH, but most probably the GSSG reduction. 

**Figure 7:**
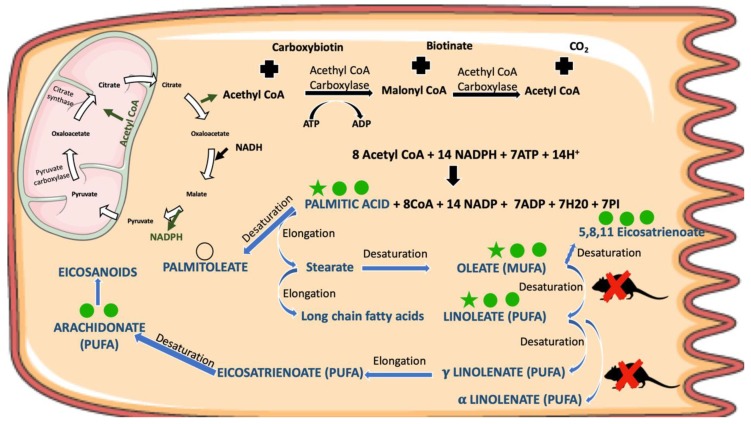
Fatty acids synthesis in the liver and the corresponding altered metabolites (VIPs >1 and *p* < 0.1) shown by multivariate analysis of GC-MS metabolomic data. The metabolites increased by nanospheres (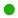) and nanostars (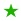); 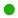 *p* value < 0.05; 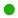 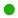 *p* value < 0.01; 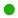 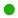 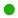 *p* value < 0.001 nanosphere-treated animals versus control; 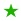 *p* value < 0.05; 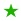 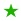 *p* value < 0.01; 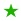 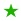 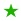 *p* value < 0.001 nanostars-treated animals versus control; or 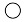 for *p* value between 0.05 and 0.1 in nanosphere-treated animals versus control.

Among the discriminant metabolites, a large group belongs to saturated (oleic, palmitic, myristic acid) and unsaturated (linoleic, palmitoleic, docosahexaenoic, arachidonic, and 5,8,11-eicosatrienoic) fatty acids. Their levels were found increased in both treatments compared with the control group and this was associated with disturbances in their biosynthesis (Figure 7). Palmitic acid represents the first product of the cytosolic fatty acids’ biosynthesis, in the presence of fatty acid synthases and NADPH [51]. It can undergo oxidation into palmitoleic acid, and elongation into stearic acid which can be further oxidized to oleic acid, considered an end-product of the de novo synthesis of fatty acid [51]. 5,8,11-Eicosatrienoic acid can be synthesized from oleic acid [52], while docosahexaenoic acid, important for brain development, from α-linoleic acid [53]. Their synthesis requires various enzymes for desaturations, elongations and beta-oxidations [53] and was related to the production of pro-inflammatory prostaglandins, cytokines, and reactive oxygen species, thus playing an important role in inflammatory diseases [54]. In fact, an important mediator of inflammatory processes, arachidonic acid, was found slightly increased in the liver of nanosphere-treated animals. On the other hand, the increase was not significant in the nanostars-treated group. Its role in the inflammatory process [55], through enzymatic oxidation with release of prostaglandins and pro-inflammatory factors was well described [56]. 

Free fatty acids are precursors of various biomolecules and they are constituents of triglycerides and glycerophospholipids [57]. Therefore their increased levels, observed in the current study, can also be a consequence of degradation or hydrolysis of these complex compounds [58]. The degradation of glycerolipids occurs in the presence of lipases, while the phospholipase A1, A2 and B are responsible for the breakdown of glycerophospholipids [58]. This can explain the high levels of linoleic acid, an essential fatty acid, that cannot be synthesized in animals as they lack the necessary enzyme for desaturation of oleic into linoleic acid [59]. Other products of triglycerides degradation, such as glycerol (nanostars treatment) and glyceric acid (both treatments), were also found increased [58]. In the nanostars-treated group, other metabolites such as serine, ethanolamine and choline, all constituents of glycerophospholipids, were also found slightly increased. As the glycerophospholipids are the main component of biological membranes, their degradation was previously related to altered cell functions, such as mitochondrial stress, apoptosis and damage of the cell membrane integrity [58]. In accordance, another metabolite, squalene, a precursor of cholesterol (essential component of cellular membrane) was found decreased in the nanostars group.

Other metabolites that discriminate between AuNPs treatments and control were uridine, uracil and inosine which are associated to the purine and pyrimidine metabolism. Uracil was found increased while its precursor, uridine, was found decreased for both AuNPs. A severe decrease was also found for inosine in both treatments. These metabolites are major energy carriers [60], crucial for the synthesis of nucleotides and for the interconversion of nucleobases, nucleosides and nucleotides [61]. Disorders of the purine and pyrimidine metabolism are associated to cellular damage [62,63] suggesting a damaging effect of both nanospheres and nanostars in the liver. 

Metabolites such as L-proline, serine, L-threonine and branched chain amino acid L-isoleucine were found slightly increased, with L-proline reaching significance (*p* value < 0.05) in the liver of nanostars-treated animals after 24 h administration versus control. It was described that rats under stress conditions, have an accelerated secretion of stress hormones which mobilize amino acids from proteins [64]. The elevated levels of the amino acids observed in this study can thus be explained by cellular responses to stress. Even more, AuNPs with anisotropic shapes were previously associated with oxidative stress and time-dependent increase in amino acids levels on tumorous A549 cells and normal 16HBE cells [65].

Overall, both nanosphere- and nanostars-treatments altered biological pathways as glutathione metabolism, protein metabolism, fatty acid metabolism, and purine and pyrimidine metabolism suggesting a similar biological response. Nevertheless, some pathways are more affected by nanostars treatment while others are more affected by nanosphere-treatment. For example, as seen in Table 4, the upregulation of several fatty acids’ synthesis (palmitic, oleic, linoleic, 5.8,11 eicosatrienoic, docoxahexaenoic) was significantly higher for nanosphere-treated animals, while increased aminoacids levels such as, L-proline, serine, L-threonine, ethanolamine and choline were found only for the nanostars-treated animals. Differences among the two treatments were also found regarding the intensity of upregulation of uracil levels and decrease in uridine and inosine belonging to purine and pyrimidine metabolism. Differences appeared also in L-lysine, an amino acid related to the lysyl-tRNA synthetases. These enzymes belong to the class of aminoacyl-tRNA synthetase necessary for charging tRNA with their associated amino acids (aminoacyl-tRNA) [62]. Therefore, even if the involved biological pathways seemed to be common to both treatments, response intensity was different, with nanospheres producing a more detrimental effect on the fatty acid synthesis while nanostars affecting more the protein synthesis.

Distinct metabolomic profiling among different types of AuNPs was already reported in the literature. For example, in an in vitro metabolomic study, HepG2 cells were exposed for 3 h to 18 nm gold nanospheres with different capping agents (citrate, poly-(sodiumsterene sulfunate) and poly-vinylpyrrolidone) [14]. Although there was a general surface-dependent decrease in intracellular metabolites for all the nanospheres, the effect was more intense for the PVP-coated AuNPs. On the other hand, citrate- and poly-(sodiumsterene sulfunate) AuNPs influenced also the ATP production in addition to the metabolomic changes. Most of the metabolites belong to the amino acid metabolism. As in our study, lipid metabolism, acyl-carnitine metabolism, carbohydrate and energy metabolism, were also pathways found altered. Nevertheless, contrary to our results, their study showed a holistic depletion of most metabolites that were attributed to the ability of AuNPs to adsorb them on their surface. As the adsorption process is related to the capping of AuNPs, the use of different capping agents in their study (citrate, poly-(sodiumsterene sulfunate) and poly-vinylpyrrolidone) versus our study (MUA) could explain the differences. Another possible explanation for the general decrease in metabolites versus general increase in our study is the use of AuNPs with different sizes (18 nm versus 40 nm) which is known to be another factor that influence biological response. Another multiomics comparison approach, including proteomics and metabolomics, was applied to the study of 5 and 30 nm gold nanospheres at 300 µM after 72 h incubation on Caco-2 cells [13]. Once again, differences were found in the metabolomic and proteomic profile between the two tested nanoparticles further associated with higher uptake of smaller nanospheres. The biological pathways were connected to amino acid and protein metabolism, glutathione metabolism, fatty acids metabolism and energy. Some of the metabolites were found increased, mainly related to the TCA cycle and energy and the vast majority, including proteins involved in DNA synthesis and repair, the synthesis of protein and the amino acid transport were found dysregulated. For 5 nm AuNPs, the biological pathways were related mainly to small molecule biochemistry, cell assembly and organization, cellular growth and proliferation, while for the 50 nm counterparts, the most affected pathways are involved in cellular compromise (degeneration) and cell morphology. Other metabolomic in vitro study using TM-4 cells exposed for 24 h to gold nanorods (10 nm width, 40 nm length) showed alteration of pathways similar to what was found in our study: the glycine, serine and threonine metabolism, cyanoamino acid metabolism, methane metabolism, aminoacyl-tRNA biosynthesis and nitrogen metabolism [66]. This corroborates the idea of a general panel of biological pathways involved in the toxicity of AuNPs. Similar pathways, as creatine, glycine, alanine, phosphocoline, UDP-NAG, pyruvate, succinate, lactate, methionine and glutathione dysregulation were found even at nontoxic concentration of AuNPs [39].

## 5. Conclusions

In conclusion, using a metabolomic approach, different results were obtained with gold nanospheres and gold nanostars in the liver. Subtle changes can be found in the levels of many molecules that precede the conventional symptoms of toxicity and conventional biochemical parameters [65]. Taking into consideration that the conventional biochemical assays are not able to distinguish between the two types of AuNPs, we can conclude that the metabolomic approach is much more sensitive and can detect slight differences in the toxicological profile, allowing the discrimination between different AuNPs at subtoxic doses.

The multivariate analysis showed that the discrimination between metabolic patterns of the liver from animals exposed to gold nanospheres versus animals exposed to gold nanostars involved metabolites such as palmitic, oleic and 5,8,11-eicosatrienoic acids, dimethylglycine, uracil inosine, uridine, L-lysine, and phosphoric acid. Among the metabolic pathways associated with the discriminated metabolites, the most significant were associated to the biosynthesis of fatty acids and the metabolism of pyrimidine and purine. This proves that metabolomics is a very useful tool for studying the effect of gold nanoparticles and should be taken into consideration as a highly sensitive alternative for comparison studies between different types of AuNPs. Future comparison studies of gold nanospheres versus gold nanostars should focus mainly in fatty acids’ synthesis and metabolism of pyrimidine and purine.

## Figures and Tables

**Table 1:** Physicochemical characterization of the gold colloidal suspensions.

	Measurement		Technique	Nanospheres		Nanostars	
Surface charge	Zeta Potential (mV)		DLS	−24.9		−27.9	
Size	Hydrodynamic diameter (nm)	Polydispersity Index	DLS	52.6 ± 0.1	0.256	64.4 ± 0.3	0.289
	Hydrodynamic diameter (nm)		NTA	59.1 ± 2.5		60.0 ± 1.5	
	Core size (nm)		TEM	40.0 ± 4.6		47.4 ± 10.7	
Concentration	AuNPs/mL		NTA	(6.68 ± 0.08) × 10^10^		(18.2 ± 0.8) × 10^10^	
	Au (µg/mL)		GFAAS	72.78 ± 4.17		180.33 ± 13.76	

**Table 2:** Gold biodistribution at 24 h after a single i.v. injection of 1.33 × 10^11^ AuNPs/kg (nanospheres or nanostars) to rats.

Tissue	Gold Concentration (ng/g)		Gold Concentration (% of the Injected Dose)		Number of AuNPs/g Organ	
	Nanospheres	Nanostars	Nanospheres	Nanostars	Nanospheres	Nanostars
Liver	3648 ± 329 ^● a bbbb^	2697 ± 205 ^bbbb^	91.2 ± 5.27 ^aaa bbb^	77.2 ± 4.7 ^aaaa bbbb^	(3.35 ± 0.30) × 10^9 a bbbb^	(2.72 ± 0.21) × 10^9 bbbb^
Spleen	2624 ± 329 ^bbb^	2271 ± 202 ^bbbb^	3.7 ± 0.4 ^bbbb^	4.3 ± 0.5 ^bbb^	(2.41 ± 0.30) × 10^9 bbb^	(2.29 ± 0.20) × 10^9 bbbb^
Lung	31.7 ± 7.0	44.0 ± 6.7	0.09 ± 0.02 ^●^	0.13 ± 0.01	(2.91 ± 0.51) × 10^7^	(4.44 ± 0.56) × 10^7^

Results are expressed as mean ± SEM using Au concentration (ng/g), % of the injected dose and number of AuNPs/kg (n = 4–5). In other organs and fluids such as heart, kidneys, brain, intestine, fat, blood, faeces and urine, Au content was lower than the limit of quantification (LOQ = 4.12 μg/kg). All statistical calculations were performed using GraphPad Prism 6 (● *p* < 0.05, ●● *p* < 0.01, ●●● *p* < 0.001; a *p* < 0.05, aa *p* < 0.01, aaa *p* < 0.001, aaaa *p* < 0.0001; b *p* < 0.05, bb *p* < 0.01, bbb *p* < 0.001, bbbb *p* < 0.0001); ● nanospheres versus nanostars; a liver versus Spleen; b liver versus Lung.

**Table 3:** Cross-validation parameters obtained for pairwise OPLS-DA models.

OPLS-DA Model	Component	R^2^X	R^2^Y	Q^2^	Permutation Tests		p (CV-ANOVA)
					R^2^	Q^2^	
Nanospheres versus control	1 + 1	0.481	0.835	0.628	0.603	−0.577	0.0053
Nanostars versus control	1 + 1	0.337	0.870	0.506	0.653	−0.494	0.0242
nanospheres versus nanostars	1 + 1	0.336	0.882	0.568	0.663	−0.566	0.0140

R^2^ represents the variation explained by the model as the sum of squares of uncorrelated (R^2^X) and predictive (R^2^Y) components; Q^2^ represents the predictive capability of the model.

**Table 4:** Liver metabolites potentially important for discrimination between various groups (nanospheres versus nanostars, nanospheres versus control, nanostars versus control) obtained from the OPLS-DA analysis.

Metabolites		Nanospheres versus Nanostars			Nanospheres versus Control			Nanostars versus Control		
		VIPs	*p* Value	Effect	VIPs	*p* Value	Effect	VIPs	*p* Value	Effect
**Fatty acids**	Oleic acid	5.35	0.0335	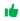	6.39	0.0012	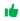 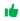	4.07	0.0324	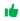
	Palmitic acid	9.62	0.0205	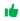	11.04	0.0013	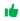 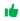	6.01	0.0232	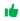
	Linoleic acid	8.58	0.1024	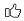	11.00	0.0015	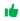 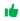	9.73	0.0116	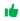
	Palmitoleic acid	3.27	0.068	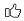	1.68	0.0708	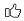	1.77	>> 0.1	-
	Docosahexaenoic acid	1.66	0.0578	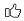	1.60	0.0010	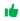 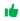 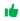	1.36	>> 0.1	-
	Arachidonic acid	2.24	0.0947	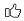	2.50	0.0031	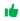 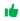	1.5	>> 0.1	-
	5,8,11-Eicosatrienoic acid	6.89	0.0354	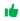	4.55	0.0120	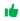	2.45	>> 0.1	-
	Myristic acid	1.04	>> 0.1	-	1.24	0.0055	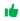 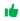	1.19	>> 0.1	-
**Organic acids and derivatives**	Squalene	-	-	-	-	-	-	1.27	0.0354	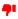
	Urea	2.15	>> 0.1	-	3.34	>> 0.1	-	6.72	0.0550	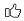
	N-Hydroxybenzamide	2.08	>> 0.1	-	2.14	0.0762	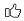	1.6	ns	-
	Glyceric acid	1.01	>> 0.1	-	2.35	0.0021	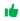 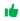	3.15	0.0021	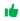 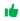
	Maleic acid	1.72	0.0853	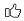	-	-	-	1.4	ns	-
	Propanoic acid	-	-	-	-	-	-	1.2	0.0255	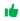
	2-Aminobenzoxazole	-	-	-	2.14	0.0456	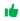	2.67	ns	-
	Glycerol	-	-	-	1.90	>> 0.1	-	3.65	0.0286	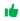
	Pyrogallol	1.07	0.1052	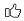	-	-	-	1.2	0.0569	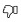
**Inorganic acids**	Phosphoric acid	7.13	0.0151	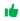	-	-	-	8.89	0.0022	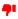 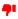
	Boric acid	1.70	0.0649	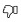	-	-	-	1.7	0.1017	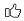
**Benzene and substituted derivatives**	o-Ethyltoluene	1.30	0.0314		1.32	>> 0.1	-	1.63	ns	-
**Aminoacids and derivatives**	L-Lysine	1.58	0.0325	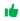	-	-	-	1.31	ns	-
	L-isoleucine	-	-	-	1.60	>> 0.1	-	2.43	0.0681	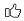
	L-proline	1.25	>> 0.1	-	2.97	>> 0.1	-	4.23	0.0104	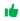
	L-serine	1.01	>> 0.1	-	3.8	>> 0.1	-	5.4	0.0737	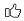
	L-threonine	2.30	>> 0.1	-	2.11	>> 0.1	-	5.02	0.0778	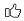
	Dimethylglicine	3.57	0.0061	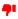 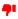	2.15	0.0941	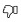	2.1	0.0610	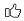
**Nucleoside/Nucleotide**	Inosine	11.4	0.0014	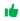 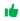	4.77	>> 0.1	-	13.04	<0.0001	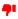 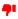 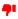 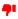
	Uridine	2.07	0.0078	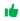 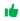	3.76	0.0296	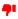	5.52	0.0016	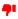 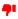
	Uracil	2.92	0.0046	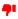 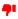	1.19	0.0637	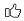	4.24	0.0003	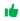 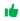 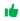
**Sugars**	Myoinositol	1.90	>>0.1	-	2.06	>> 0.1	-	2.45	0.0709	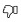
	D-Psicofuranose	1.16	>>0.1	-	2.08	>> 0.1	-	3.01	0.066	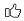
**Unknown**	Unknown 1	1.67	0.0022	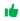 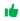	-	-	-	1.2	>> 0.1	-
	Unknown 2	11.35	0.0076	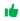 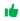	10.37	>> 0.1	-	2.58	>> 0.1	-
	Unknown 3	2.50	0.0045	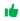 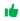	1.39	>> 0.1	-	1.11	>> 0.1	-

For all discriminant metabolites VIPs > 1; *p* values between groups (nanospheres versus nanostars; nanospheres versus control, nanostars versus control) were calculated by the unpaired Student´s t test (data normality distributed) and the unpaired Mann-Whitney test (data non-normally distributed). Significance was considered at *p* value < 0.05. The metabolites (*p* value < 0.05) upregulated are marked with 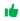 and downregulated marked with 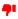 or with 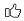 and 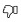 (*p* value between 0.05 and 0.1); - discriminant not found in the comparison; ns: not statistically different.

## Supplementary Materials

The following are available online at https://www.mdpi.com/2079-4991/9/11/1606/s1. Figure S1: Representative scheme of the experimental design. A multiapproach study of the effect of ~40 nm MUA-coated Au nanospheres and nanostars, 24 h after i.v. administration to Wistar rats, Table S1: Parameters used in the MZmine software for the data pre-processing for the liver intracellular metabolome analysis, Table S2: The identification of liver intracellular metabolites from OPLS-DA analyze as being potentially important for discrimination (Spheres vs control, Stars vs control, Spheres vs Stars).
