# Supplementary material for: A Metabolomic Approach for the In Vivo Study of Gold Nanospheres and Nanostars after a Single-Dose Intravenous Administration to Wistar Rats
Source: Nanomaterials (Basel). 2019 Nov 12;9(11):1606. doi: 10.3390/nano9111606 (PMC6915599; doi:10.3390/nano9111606)
Supplement: Supplementary file 1 [file nanomaterials-09-01606-s001.pdf]

nanomaterials

Article

A Metabolomic Approach for the In Vivo Study of Gold Nanospheres and Nanostars after a Single-Dose Intravenous Administration to Wistar Rats

Maria Enea [1,2,*], Ana Margarida Araújo [1], Miguel Peixoto de Almeida [2], Maria Elisa Soares [1], Salomé Gonçalves-Monteiro [3], Paula Guedes de Pinho [1], Eulália Pereira [2,*], Maria de Lourdes Bastos [1] and Helena Carmo [1,*]

1 UCIBIO/REQUIMTE, Laboratory of Toxicology, Faculty of Pharmacy, University of Porto, 4050-313 Porto, Portugal; ana.margarida.c.araujo@gmail.com (A.M.A.); mematos25@gmail.com (M.E.S.); pguedes@ff.up.pt (P.G.d.P.); mlbastos@ff.up.pt (M.d.L.B.)
2 LAQV/REQUIMTE, Department of Chemistry and Biochemistry, Faculty of Sciences, University of Porto, 4169-007 Porto, Portugal; mpda@fc.up.pt (M.P.d.A.)
3 LAQV/REQUIMTE, Department of Drug Sciences, Laboratory of Pharmacology, Faculty of Pharmacy, University of Porto, 4150-755 Porto, Portugal; salomegmonteiro@hotmail.com
* Correspondence: maria.enea@fc.up.pt (M.E.); eulalia.pereira@fc.up.pt (E.P); helenacarmo@ff.up.pt (H.C)

1. Experimental design

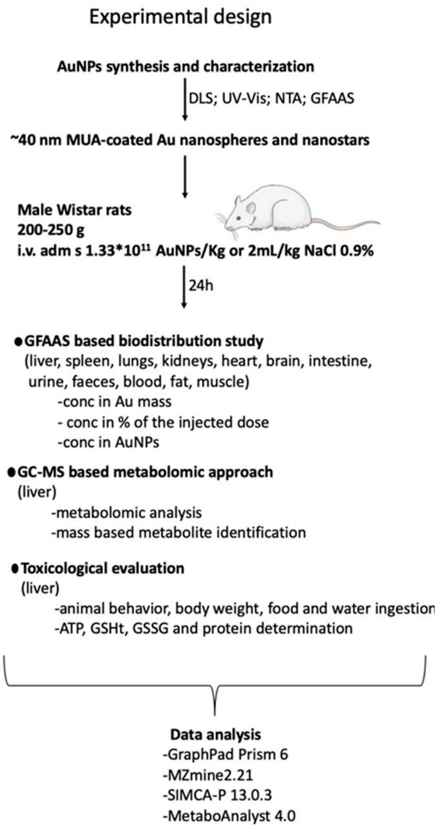

Figure A1. Representative scheme of the experimental design. A multiapproach study of the effect of ~40 nm MUA-coated Au nanospheres and nanostars, 24 h after i.v. administration to Wistar rats.

2. Characterization of AuNPs

The synthesized AuNPs were characterized using UV-Vis spectrophotometry, transmission electron microscopy (TEM), Dynamic Light Scattering (DLS) and Nanoparticle Tracking Analysis

(NTA). The UV-Vis spectra were measured in a range of 300 -1000 nm, at room temperature using a UV-Vis spectrophotometer Varian Cary 50 Bio UV-Visible (Agilent Technologies, Santa Clara, CA, US) in a quartz cell (Starna). Images of the synthesized nanoparticles samples were obtained using a Hitachi H-8100 transmission electron microscope (Hitachi, Tokyo, Japan) operated at 200 kV and the ImageJ software (http://rsbweb.nih.gov/ij/) was applied to calculate the average core diameter and the standard distribution from minimum 100 nanoparticles. The hydrodynamic diameter and zeta potential of the AuNPs were measured by Dynamic Light Scattering (DLS) measurements of size and then zeta potential determinations were performed using a Zetasizer Nano ZS (Malvern Panalytical, Malvern, UK), with a 4 mW He-Ne laser (633 nm), at 25 ºC using disposable capillary cells. The measurements were taken as the average of a minimum of three runs, each containing 11-submeasurements and the results are presented as mean z-average (PSD) together with the polydispersity index (PDI). The NTA measurements of the hydrodynamic diameter and of AuNPs concentrations were performed using a NanoSight NS300 instrument (Malvern Panalytical, Malvern, UK) and 50x or 100x diluted (with 10 mM phosphate buffer, pH 7.4) samples. The results are presented as Mean+/- Standard error of the mean.

3. Quantification of gold in biological samples by Graphite Furnace Atomic Absorption Spectrometry (GFAAS)

The content in gold was analyzed by GFAAS in whole blood, faeces, urine, liver, spleen, lung, heart, kidney, brain, small intestine (duodenum), fat, skeletal muscle (quadriceps femuris) and tail. Briefly, the samples were first digested with a mixture of 5% HNO3 and 30% H_2O_2 (4:1) at 105 ºC and further analysed using an AAnalyst 600 Atomic Absorption Spectrophotometer (Perkin-Elmer, Shelton, CT, USA) equipped with an auto-sampler. A previous 1:50 dilution with 0.2% HCl was necessary for some of the biological samples. Using an autosampler, 20 µL of standard solution/diluted sample and 10 µL of matrix modifier [3 g/L Pd(NO3)2 + 2 g/L Mg(NO3)2] were injected into the graphite furnace to measure the Au content. All analyses were conducted at 242.8 nm and the Au atomized at 1800 ºC. The readings were taken by using the peak area.

4. Target organ metabolome analysis

4.1. Chromatographic and mass spectrometry settings

The chromatographic analysis was performed using an EVOQ 436 GC system (Bruker Daltonics, Fremont, CA) coupled to a SCION Triple Quadrupole mass detector, using a capillary column Rxi-5Sil MS (30 m*0.25 mm*0.25 µm) from RESTEK. Helium C-60 (Gasin, Portugal) was used as the carrier gas at a constant flow rate of 1.0 mL/min. Samples (2 µL) were injected in split mode (ratio 1:20) and the injector temperature was 250 ºC (held for 20 min). The oven temperature was fixed at 70 ºC for 2 min, then increasing to 250 ºC (rate 15 ºC/min), held for 2 min, then increasing to 300 ºC (rate 10 ºC/min) and held for 5 min. Total separation run time was 26 min. The MS detector was operated in EI mode (70 eV). The transfer line temperature was 280 ºC, manifold temperature was 40 ºC and the EI temperature was 270 ºC. Data acquisition was performed in full scan mode with a mass range between 50 and 400 m/z. A QC sample was repeatedly analyzed under the same conditions, one every nine samples, to guarantee the analytical reproducibility of the method. Sample preparation and GC-MS acquisition were randomized to avoid analytical bias.

Table A1. Parameters used in the MZmine software for the data pre-processing for the liver intracellular metabolome analysis.

Parameter		Liver
**Crop filtering**	m/z	50-400
	RT (min)	4-25
Peak detection	Noise level	8*10^4
Chromatogram deconvolution	Peak range (min)	0.02-0.3
	Baseline level	5*10^5
Alignment	m/z tolerance	0.5
	RT tolerance (min)	0.05

Table A2. The identification of liver intracellular metabolites from OPLS-DA analyze as being potentially important for discrimination (Spheres vs control, Stars vs control, Spheres vs Stars).

Metabolite	RT(min)	m/z	RIlit	RIcalc	R Match	Identification method
Ascorbic acid 4TMS	13.171	73+147	1971	1930	845	NIST
Asparagine 3TMS	11.203	73+116	1670	1653	835	NIST
Arachidonic acid TMS	16.634	73	2383	2383	818	STD
o-Ethyltoluene	4.547	105+120	970	974	898	STD
Benzohydroxamic acid	9.668	73+147	1496	1464	639	NIST
Boric acid 3TMS	4.624	73	1010	981	717	STD
Cholesterol TMS	23.216	73+129	3150	3150	908	STD
Dimethylglicine 3TMS	4.655	58	1001	984	843	NIST
Desmosterol TMS	23.625	69+73	3202	3189	894	STD
Docosahexaenoic acid TMS	18.161	73+75	2562	2550	775	NIST
Erythrono-1,4-lactone 2TMS	8.774	73+147	1310	1365	841	NIST
Fumaric acid 2TMS	8.527	73+245	1353	1339	911	NIST
Glycerol 3TMS	8.982	73+204	1300	1387	798	NIST
Glycerol monostearate 2TMS	19.974	73	2806	2767	873	NIST
Glyceric acid 3TMS	8.311	73	1344	1316	871	NIST
Glycine 2TMS	6.175	102	1105	1115	931	STD
Inosine 4TMS	18.238	73+217	2562	2559	858	NIST
Lactic acid 2TMS	6.434	73	1066	1051	949	NIST
L-Alanine 2TMS	5.951	116	1095	1096	918	STD
L-Glutamic acid 3TMS	10.81	73+246	1629	1604	947	STD
L-Glutamine 3TMS	11.975	73+156	1768	1757	876	NIST
L-Isoleucine 2TMS	7.987	73+158	1301	1283	900	NIST
L-Lysine 4TMS	13.009	73	1801	1905	766	STD
L-proline 2TMS	8.056	73+142	1305	1289	893	STD
L-Threonine 3TMS	8.835	73	1367	1372	911	STD
L-Valine 2TMS	7.2	73+144	1224	1206	904	STD
L-5-Oxoproline 2TMS	10.054	73+147+156	1522	1511	898	STD
Linoleic acid TMS	15.007	67+73+75	2212	2202	927	STD
Maleic acid 2TBDMS	11.643	73	1740	1710	733	NIST
Malic acid 3TMS	9.772	73+147+233	1390	1476	908	STD
Maltose	19.426	73+204	2693	2698	935	NIST
Myo-Inositol 6TMS	14.112	73+147	2113	2076	911	STD
Myristic acid TMS	12.53	73+117	1850	1836	862	NIST
Oleic acid TMS	15.053	73+75+117	2218	2207	921	STD
Palmitoleic acid TMS	13.718	73	1995	2014	859	NIST
Palmitic acid TMS	13.842	73+117+132	2050	2033	920	STD
Phosphoric acid	11.843	73+299	1741	1739	763	NIST
Phosphoric acid 3TMS	7.763	73+299	1286	1261	915	STD
Propanoic acid 3TMS	5.303	73+174	1063	1040	859	NIST
Pyrogallol 3TMS	10.679	73	1557	1587	737	NIST
Serine 3TMS	8.604	73+204	1368	1347	867	STD
Stearic acid TMS	15.253	73+75	2246	2230	932	NIST
Squalene	20.314	69+81	2832	2810	818	NIST

Uracil 2TMS	8.411	73+99	1343	1327	867	NIST
Urea 2TMS	7.532	147+73+189	1249	1239	931	STD
Uridine 3TMS	17.166	73	2469	2439	854	NIST
2-Aminobenzoxazole 2TMS	10.224	73+75+147	1511	1532	722	NIST
5,8,11-Eicosatrienoic acid TMS	16.441	73+75	2371	2362	781	NIST
3- Hydroxibutiric acid 2TMS	6.599	73+147	1167	1153	897	NIST
7H-Ppurine	13.633	73+147+353	2011	2001	889	NIST
9H-Purin-6-ol 2 TMS	12.252	73+265	1811	1796	870	NIST
2,3,4,5.Tetrahydroxypentanoic acid-1,4-lactone 3TMS	11.458	73	1662	1686	734	NIST
3-α-Mannobiose	19.812	73	2749	2746	864	NIST
α-D-Mannopyranose 5TMS	12.168	73+204	1794	1784	884	NIST
β-Hydroxypyruvic acid 3TMS	6.267	73	1115	1123	760	NIST
β-D-Talopyranose 5TMS	12.878	73+204	1896	1886	849	NIST
D-(+)-Cellobiose	19.604	73+204	2708	2720	828	NIST
D-Galactose 2,3,4,5,6-pentakis	12.955	73+147	1898	1897	887	NIST
D-Glucose 2,3,4,5,6-pentakis	12.738	73+147	1883	1866	902	STD
D-Mannitol 6TMS	13.279	73	1958	1947	766	NIST
D-Mannopyranose 5TMS	12.792	73+204	1890	1874	926	NIST
D-Psicofuranose 5TMS	12.576	73+217	1837	1843	833	NIST
D-Tagatofuranose 5TMS	12.33	73	1801	1807	789	NIST
Unknown 1	14.804	73+204	-	2174	-	-
Unknown 2	14.945	73+147	-	2193	-	-
Unknow 3	17.02	73	-	2423	-	-
Unknown 4	16.179	73+147	-	2334	-	-
Unknown 5	17.02	73+302	-	2423	-	-

[1] The identification was made using the retention time at peak apex (RT), characteristic ions (m/z), retention index from the literature (RIlit) and calculated (RIcalc), reverse and forward values of match from NIST library (2014). VIPs >1; STD- metabolites that were confirmed using standards injected in GC-MS.

Funding: This research was funded by European Union (FEDER funds POCI/01/0145/FEDER/007728, POCI-01-0145-FEDER-029584, POCI/01/0145/FEDER/007265) and National Funds (FCT/MEC, Fundação para a Ciência e a Tecnologia and Ministério da Educação e Ciência) under the Partnership Agreement PT2020 UID/MULTI/04378/2013 (UCIBIO-REQUIMTE), UID/QUI/50006/2019 and UID/QUI/50006/2013 (LAQV-REQUIMTE). It was financed also through NANOMODE project (PTDC/CTM-NAN/30589/2017). M.E and A.M.A acknowledge the national funds (FCT) for their doctoral grants PD/BD/109634/2015 (PDQS) and SFRH/BD/107708/2015.

Acknowledgments: In this section you can acknowledge any support given which is not covered by the author contribution or funding sections. This may include administrative and technical support, or donations in kind (e.g., materials used for experiments).

Conflicts of Interest: The authors declare no conflict of interest.

© 2019 by the authors. Submitted for possible open access publication under the terms and conditions of the Creative Commons Attribution (CC BY) license (http://creativecommons.org/licenses/by/4.0/).
